# Ontology- and LLM-based data harmonization for federated learning in healthcare

**DOI:** 10.3389/fdgth.2026.1756555

**Published:** 2026-03-18

**Authors:** Natallia Kokash, Lei Wang, Thomas H. Gillespie, Adam S. Z. Belloum, Paola Grosso, Sara Quinney, Lang Li, Bernard de Bono

**Affiliations:** 1 Institute of Informatics, University of Amsterdam, Amsterdam, Netherlands; 2 College of Medicine, The Ohio State University, Columbus, OH, United States; 3 Department of Neuroscience, University of California, San Diego, CA, United States; 4 School of Medicine, Indiana University, Indianapolis, IN, United States

**Keywords:** biomedical ontologies, data harmonization, federated learning, healthcare, LLM application

## Abstract

**Introduction:**

Semantic heterogeneity across electronic health records (EHRs) limits scalable and privacy-preserving analytics in healthcare. While federated learning (FL) enables collaborative modeling without sharing raw data, it requires consistent, ontology-aligned representations. We present an ontology- and large language model (LLM)-based data harmonization approach to support secure, interoperable FL workflows.

**Methods:**

We propose a general two-step pipeline for converting or annotating clinical text into a predefined target ontology format. First, candidate concepts are retrieved from the target vocabulary using embedding-based similarity search or ontology cross-references. Second, an LLM acts as a semantic validator, accepting or rejecting candidates based on explicit equivalence or subsumption criteria. The approach is ontology-agnostic and configurable; mapping to MONDO and HPO is demonstrated as a real-world use case. Final accepted mappings were evaluated against independent human expert assessment.

**Results:**

Across two clinical datasets, expert-LLM agreement reached up to 92%, with overall performance ranging from 78% to 91% depending on candidate-generation strategy. Retrieval alone was insufficient for reliable mapping, whereas LLM-based validation substantially improved precision while complementary retrieval strategies improved recall.

**Discussion:**

The proposed pipeline transforms ontology-based harmonization from a manual expert task into a reusable and configurable workflow suitable for federated healthcare research. By combining high-recall retrieval with LLM-based semantic adjudication, the approach enables scalable, privacy-preserving conversion of heterogeneous clinical text into standardized representations across domains.

## 1 Introduction

The rapid digitalization of healthcare has led to the proliferation of electronic health records (EHRs), offering unprecedented opportunities for data-driven medical research and clinical decision-making. However, leveraging this data at scale remains challenging due to stringent privacy regulations, security risks, and ethical concerns associated with centralized data storage. Traditional machine learning (ML) approaches rely on the aggregation of patient data into centralized repositories, making them more vulnerable to data breaches and creating additional challenges in aligning with the principles of regulations such as the Health Insurance Portability and Accountability Act (HIPAA) in the United States [1] and the General Data Protection Regulation (GDPR) in Europe [2]. To address these concerns, federated learning (FL) has emerged as a promising paradigm for collaborative model training without the need for direct data sharing [3].

In FL, multiple organizations contribute to the training of a *global model* without transferring raw data across institutional boundaries. The global model is a single ML model whose parameters are iteratively optimized using distributed data. A central coordinator initializes the model and orchestrates a sequence of training rounds. In each round, the current version of the global model is sent to participating institutions, where it is updated using local data through one or more steps of local optimization. Instead of sharing data, institutions return only model updates (e.g., gradients or updated parameters) to the coordinator. These updates are aggregated to produce an improved version of the global model for the next round [4]. By keeping patient-level data within institutional boundaries, this approach reduces data transfer risks and supports compliance with the data minimization and purpose limitation principles underlying privacy regulations such as GDPR and HIPAA.

Despite apparent advantages, applying FL in healthcare presents significant challenges, including data heterogeneity, security vulnerabilities, and computational overhead [5, 6]. A major obstacle is the alignment and harmonization of heterogeneous electronic health record (EHR) formats, which vary substantially across institutions due to differences in clinical terminologies, data standards, and infrastructure [7].

*Data harmonization* is the practice of “reconciling” various types, levels and sources of data in formats that are compatible and comparable, and thus useful for better decision-making [8]. Data harmonization often relies on probabilistic and/or ML-based entity resolution techniques. Schema matching automates the identification of correspondences between fields in different datasets, such as aligning “DOB” in one database with “DateOfBirth” in another. Modern tools leverage natural language processing (NLP) and ontology-based reasoning to improve accuracy [9]. Type conversion ensures consistent representation of data types, such as converting blood pressure values stored as strings into standardized numeric formats or translating medication codes between vocabularies like RxNorm [10]—a standardized nomenclature for clinical drugs maintained by the U.S. National Library of Medicine—and SNOMED CT [11]—a comprehensive clinical terminology to encode diagnoses, symptoms, and procedures in EHRs. In healthcare, these automated techniques support critical applications like patient cohort identification, population health monitoring, and real-time clinical decision support, reducing manual curation and improving data quality for ML pipelines and interoperable health information systems [12].

Large language models (LLMs), trained on vast corpora of biomedical literature and structured clinical data, have demonstrated strong capabilities in natural language understanding and information extraction [13]. They can be leveraged to standardize disparate EHRs, align ontologies, and mitigate discrepancies in medical coding practices across different hospitals and research centers. However, ensuring the trustworthiness, bias mitigation, and interpretability of LLMs in clinical applications remains a critical research frontier [14].

This paper explores the intersection of FL and healthcare, focusing on data harmonization strategies within privacy-preserving data access environments. With attention to security and regulatory compliance challenges, we are working on the integration of LLM-based functionality to a programmable FL framework to enable healthcare data alignment. We show how our two-step ontology- and LLM-based data alignment strategy was instrumental in the mapping of healthcare data for a real-world project. In the first step, a candidate-generation module—which we refer to as the *converter*—produced matching pairs using (a) vector-space embeddings [15] and/or (b) ontology-based cross-reference matching. In the second step, an LLM was used to accept or reject each candidate pair.

## 2 Methods

### 2.1 Federated learning frameworks

Our approach builds on two existing FL frameworks—Vantage6 [16] and Brane [17]—which together provide the infrastructure on which our contributions are layered. We describe them here as background context.

**Figure 1:**
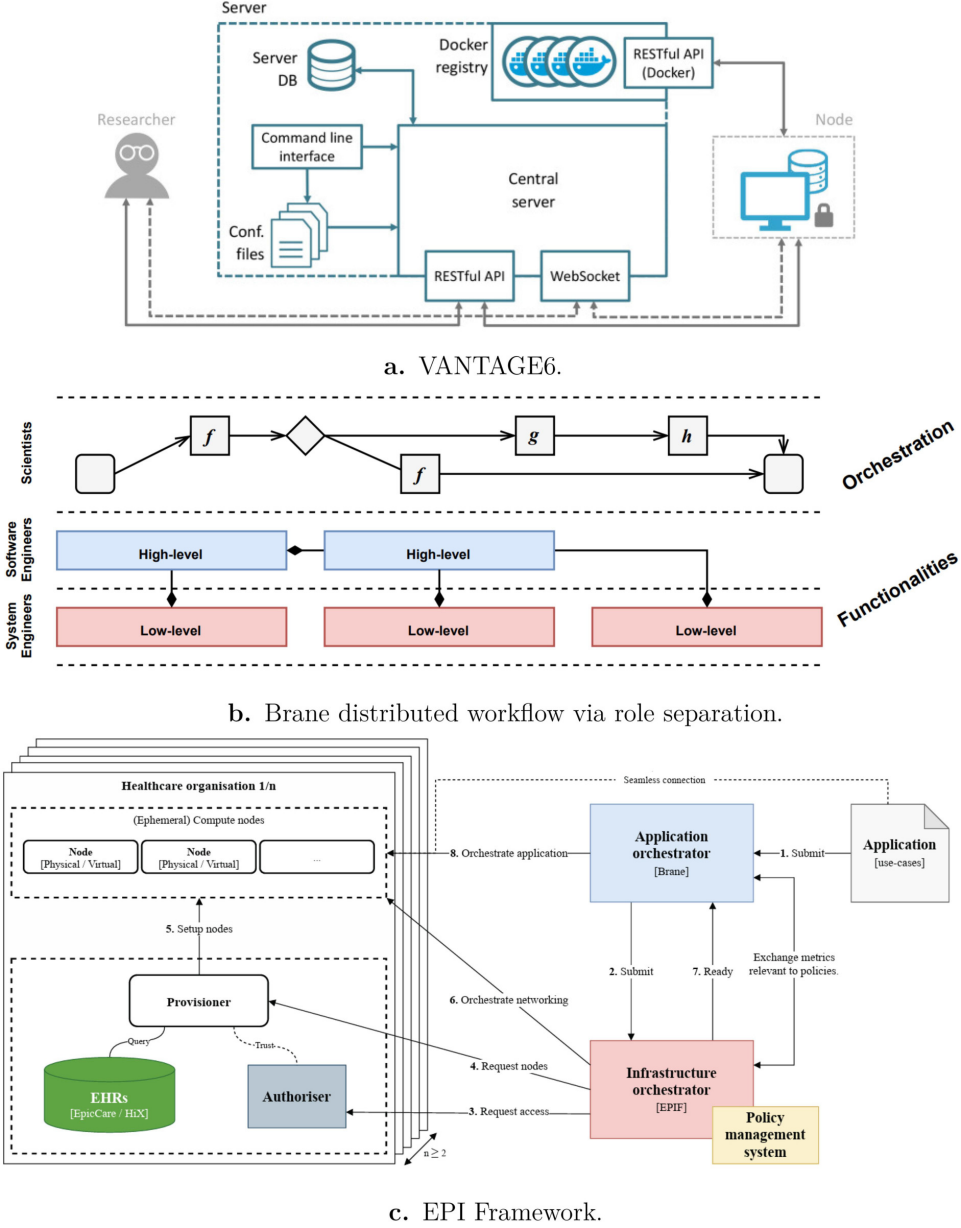
Distributed workflow and federated execution frameworks: **(a)** VANTAGE6 server architecture. Reproduced from “VANTAGE6's server. An administrator uses the command line interface to configure and start the server. After the server loads its configuration parameters (which are stored in a YAML file), it exposes its RESTful API. It is worth noting that the central server's RESTful API is different from that of the Docker registry” by Arturo Moncada-Torres, Frank Martin, Melle Sieswerda, Johan Van Soest and Gijs Geleijnse, Public Domain Mark 1.0 Universal, **(b)** Brane role-based workflow separation. Reproduced from “Brane's separation of concerns follows the roles and layers of the technical stack” by Onno Valkering, Reginald Cushing and Adam Belloum, licensed under CC BY. **(c)** EPI framework design. Reproduced from “A high level view of the different EPIF components and their interactions after an application request” by Jamila Alsayed Kassem, Onno Valkering, Adam Belloum and Paola Grosso, licensed under CC BY.

Vantage6 [16] allows researchers to perform ML operations on client’s data located at worker (computing) nodes as shown in Figure 1a. The process is orchestrated by a server (central node). A researcher can submit a task to the server with an algorithm and input parameters. The algorithm is first implemented using Vantage6 tools and built into a Docker image [18]. After a task is submitted to the server, the server sends the task information to a computing node. A computing node automatically detects the server, gets the task information, and executes the algorithm on local data. The intermediate results are sent back to the server for aggregation, and the iterative process of FL repeats to update the global model. The final result is sent back to the researcher when the computation is complete.

Brane is a programmable framework for secure data exchange and scientific workflow orchestration. The primary purpose of Brane is to support efficient and secure data exchange among research organizations [17]. Brane utilizes containerization to encapsulate functionalities as portable building blocks. Application orchestration can be expressed using an intuitive domain-specific language or user-friendly interactive notebooks. End users with limited programming experience are empowered to compose workflows without the need to deal with the underlying technical details.

A key principle of Brane’s design is the clear separation of concerns based on specialized user roles, as shown in Figure 1b: (i) *domain scientists* focus on data analysis without managing execution details, (ii) *software engineers* develop and optimize data processing workflows, (iii) *systems engineers* maintain the infrastructure and ensure system efficiency.

The only requirement for remote resources is to be able to install and run containers. The runtime system can automatically convert packages [Open Container Initiative (OCI) images [20]] to the appropriate container image format. By default, direct access to resources is assumed; when this is not possible (e.g., not permitted by participating organizations or regulatory policies), an optional indirection layer is enabled.

Brane is a key component of the Enabling Personalized Interventions (EPI) framework [21]. The EPI framework provides a secure, distributed data platform that supports personalized health insights through analytics and decision support tools. Its main features are:

- Allowing analysts to process data across multiple organizations without dealing with technical complexities.
- Enforcing user-defined data policies during all stages of data processing.

Figure 1c shows the main components of the framework, which are:

- the *orchestrators* (both at the application level and infrastructural level);
- the *policy management system*;
- the components required to be present at the participating institutions: the *resource provisional* and the *authorizers*.

At the network level, the EPI platform enforces security and low-level policies to protect data sharing.

### 2.2 AI assisted open FL frameworks

A proof-of-concept collaborative network was successfully deployed with St. Antonius Ziekenhuis, UMC Utrecht, and Princes Maxima Centrum, processing patient data across two hospitals and the Dutch supercomputer center SURF [21]. While this validated the EPI framework for privacy-sensitive healthcare research, the deployment operated within a tightly scoped closed consortium in which partners agreed on research goals, data content, and compliance expectations upfront. Because the FL scope, data schemata, and organizational conventions are known in advance, workflow design is considerably more tractable than in open-ended scenarios. However, this also means the resulting workflows are rarely reusable: they are built around a priori knowledge of the participating datasets and are difficult to reproduce even for structurally identical projects. Moreover, existing FL platforms typically leave data alignment to application-level developers, imposing technical barriers that domain experts alone cannot easily overcome.

A key strength of the Brane framework is its flexibility. It enables secure private data access and solution development, and—given sufficient support infrastructure—has potential comparable to enterprise low-code application platforms [22], a rapidly growing global market [USD 24.83 billion in 2023, projected to reach USD 101.68 billion by 2030 [23]].

We therefore aim to adapt Brane/EPI to support open consortia of healthcare organizations interested in on-demand FL networks. In our vision, a scientist (i) designs a high-level workflow and, (ii) together with engineers, deploys a project server that allows organizations with relevant data to (iii) join via a pre-configured container. The scientist then (iv) distributes ML algorithm images for local execution and (v) aggregates returned results on the server.

The combination of Vantage6, Brane, and EPI provides technical components for this vision. Although Brane is not a dedicated FL platform, its programmable and general-purpose nature makes it suitable for FL workflows. Liu [24] demonstrated deployment of Vantage6 algorithm images on a Brane network, identifying the lack of data converters as a key obstacle to scalable FL workflows.

The main challenge remains the absence of ready-to-use resources and community support to keep FL workflow design truly “low-code.” While integrating Python-based ML libraries [e.g., PyTorch [25]] within Brane is straightforward, regulatory compliance and data harmonization still require application-level solutions.

**Novel contribution.** We address the data harmonization gap by integrating LLM-based assistants and agents into Brane/EPI, introducing data-agnostic conversion functions and evaluating their application to mapping patient EHRs to standardized biomedical vocabularies.

### 2.3 Aligning biomedical data via ontologies

#### 2.3.1 Background: biomedical ontologies

The biomedical domain uses a rich ecosystem of ontologies to standardize diseases, diagnoses, treatments, laboratory findings, and clinical outcomes. The ontologies most relevant to this work are SNOMED CT [11], ICD-10 [26], MONDO [27], and HPO [28]. ICD-10, maintained by the WHO, is the global standard for disease classification used in over 150 countries. HPO standardizes human phenotype descriptions, widely adopted in genomic diagnostics and rare disease research. MONDO integrates multiple disease ontologies—including Orphanet, OMIM, and ClinGen—to unify rare disease research. Table 1 summarizes the full set of ontologies referenced in this work.

**Table 1:** Biomedical ontologies.

Name	Purpose	Key features	Use cases
SNOMED CT	Standardized clinical terminology for EHRs	Provides hierarchical relationships and standardized codes for diseases, symptoms, and procedures	Clinical documentation, decision support, interoperability in healthcare IT systems
ICD-10	Global classification of diseases, maintained by WHO	Provides alphanumeric codes for diseases and health conditions	Public health monitoring, insurance claims, medical record standardization
MONDO	Unified disease ontology integrating multiple sources	Harmonizes data from Orphanet, OMIM, and DOID	Research in rare diseases, precision medicine, biomedical data integration
HPO	Standardized vocabulary for human disease phenotypes	Describes phenotypic abnormalities in a hierarchical structure	Clinical genomics, rare disease research, computational phenotyping
ORDO	Orphanet Rare Disease Ontology, European database for rare diseases and orphan drugs	Disease and gene information	Used in rare disease research, clinical guidelines, regulatory agencies
OMIM	Online Mendelian Inheritance in Man, a catalog of human genes and genetic disorders	Provides gene-disease relationships, clinical descriptions, inheritance patterns	Used in clinical genetics, genomic research, and precision medicine
ClinGen	Clinical Genome Resource, NIH database of clinically relevant genetic variants	Assists in variant classification for genetic diagnostics	Supports clinical diagnostics, personalized medicine, and regulatory decisions
EBI	Bioinformatics resource for genomics, proteomics	Provides large-scale biological data	Used in genomics, pharmacology, and data integration
MedGen	Clinical genetics database	Organizes human genetic conditions	Used in genetic counseling, rare disease research
MeSH	Standardized biomedical terminology	Indexes PubMed, MEDLINE, and biomedical databases	Used in literature indexing and biomedical research
UMLS	Unified medical terminology system	Maps multiple vocabularies for interoperability	Used in clinical decision support, EHRs, NLP
OBO	Collection of interoperable ontologies	Supports semantic consistency in biological data	Used in bioinformatics and AI-driven research
DOID	Standardized disease classification	Links genetic and environmental factors in diseases	Used in genomics, precision medicine, and disease annotation
NCIT	Oncology-specific vocabulary	Standardizes cancer terminology	Used in clinical trials, cancer research, and informatics
RxNorm	Standardized drug terminology maintained by the U.S. National Library of Medicine	Provides normalized names and unique identifiers for clinical drugs, linking various national drug terminologies	Used in electronic health records (EHRs), clinical decision support, and pharmacy systems
ATC	Anatomical Therapeutic Chemical Classification System, classifies drugs based on mechanism of action and therapeutic use	Hierarchical classification of drugs into five levels, covering active ingredients and therapeutic groups	Used in pharmacoepidemiology, drug regulation, and healthcare analytics
MedDRA	Medical Dictionary for Regulatory Activities, terminology for medical conditions, adverse events, and drug safety monitoring	Maintains hierarchical coding for diseases, symptoms, and adverse drug reactions	Used in pharmacovigilance, clinical trials, and regulatory reporting

These ontologies differ in focus, granularity, and intended use: some target clinical documentation (SNOMED CT, ICD-10), others genetic research (HPO, ClinGen, OMIM), and others pharmacology [RxNorm [10], ATC [29], MedDRA [30]]. Because medical knowledge evolves continuously, multiple regional versions coexist, and even widely used standards such as SNOMED CT and ICD-10 undergo frequent updates [31], making full interoperability a persistent challenge. Existing harmonization initiatives, e.g., the OHDSI Common Data Model, the LOINC-SNOMED harmonization initiative, and the UMLS Metathesaurus, address this partially, but full automation remains an open problem requiring advances in ontology alignment, ML-driven entity resolution, and expert validation.

#### 2.3.2 Our contribution: a two-step LLM-based alignment pipeline

**Figure 2:**
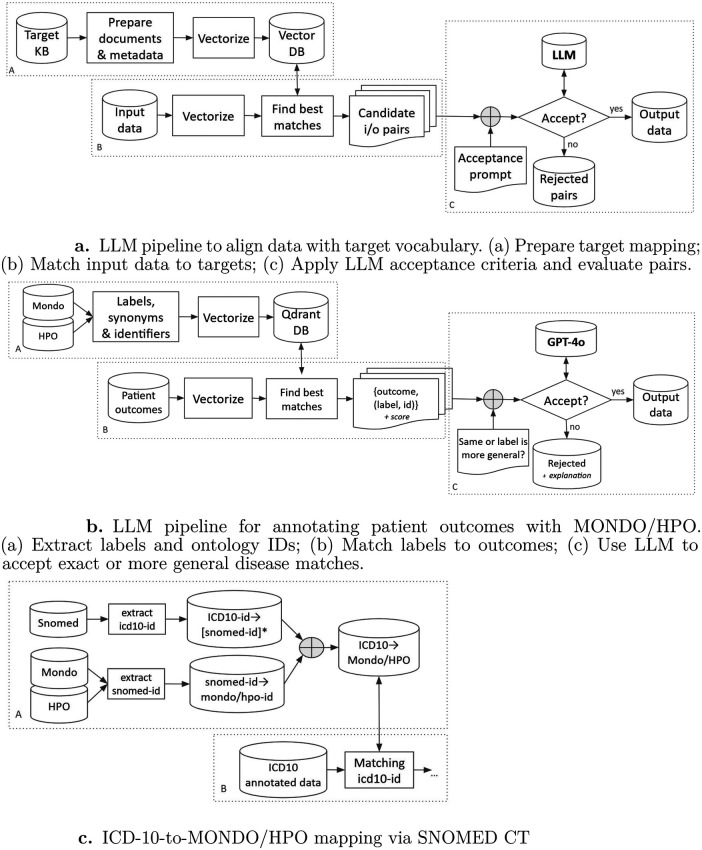
LLM-based pipelines for ontology-aware data alignment: **(a)** general vocabulary alignment workflow, **(b)** instance-level outcome annotation with MONDO/HPO. **(c)** candidate pair generation via SNOMED CT.

To address this automation gap, we propose a generic two-step pipeline for mapping unannotated or differently-annotated EHR data to a target biomedical ontology (Figure 2a). Both steps are novel integrations within the Brane/EPI framework rather than applications of off-the-shelf tools.

In **Step 1** (candidate generation), we enable Retrieval Augmented Generation (RAG) [32] over the target vocabulary: ontology labels and synonyms are embedded into a vector store, and for each input record the nearest-neighbour search retrieves the best-matching candidate terms. This produces a set of *(input, candidate)* pairs for review.

In **Step 2** (LLM-based acceptance), an LLM evaluates each candidate pair against explicitly defined acceptance criteria and accepts or rejects it. Crucially, the acceptance criteria are formulated as a natural-language prompt, making the pipeline adaptable to different ontological relations (e.g., equivalence, subsumption) without code changes. The same two-step design handles both unannotated text and pre-coded data, as described in Section 2.4.

Annotating EHR observations with ontology terms enables patient cohort selection and creation of harmonized datasets for FL training. Figure 2a illustrates the generic form of our two-step pipeline; the specific instantiation for our EHR harmonization project context is shown in Figure 2b.

### 2.4 Application to the MPRINT use case

The Maternal and Pediatric Precision in Therapeutics (MPRINT) hub aggregates, presents, and expands the available knowledge, tools, and expertise in maternal and pediatric therapeutics to the broader research, regulatory science, and drug development communities [33]. The MPRINT working group processes data from multiple healthcare organizations, spanning patient demographics, diagnoses, medications, procedures, laboratory tests, and diagnostic images. We applied the two-step pipeline to align textual EHR data for a MPRINT drug reporting study aimed at establishing relationships between medication or chemical exposures during pregnancy and their effects on pregnancy, postpartum, and newborn health.

We focus here on mapping pregnancy outcome descriptions to MONDO and/or HPO. MONDO and HPO are complementary: MONDO standardizes disease definitions, while HPO defines phenotypic abnormalities. They are linked through annotations, so using labels from both ontologies improves mapping recall. In an *ontological annotation task*, *precision* is the proportion of predicted ontology terms that are correct, and *recall* is the proportion of relevant terms successfully predicted [34].

As shown in Figure 2b, the pipeline proceeds in two steps. Step 1 constructs a semantic index over the target ontology terms and queries it for each input record; Step 2 submits the retrieved candidates to an LLM for acceptance filtering.

#### 2.4.1 RAG-based candidate generation

We construct the index from both MONDO and HPO, which are provided in JSON graph format containing nodes that represent controlled vocabulary terms. For each node whose identifier carries the prefix MONDO_ or HP_, we extract the primary label (lbl) and all available synonyms from the node metadata. Descriptions, definitions, and hierarchical relations are not used.

Embeddings are generated at the *term level*: each primary label and each synonym is embedded independently using the OpenAI text-embedding-ada-002 model, which produces dense vectors of dimensionality d=1536. Formally, for an input text string t (a label or synonym):

$$v=f_{\text{ada-002}}(t),\;v\in R^{1536}.$$

A single ontology concept therefore yields a family of embedding vectors:

$$\{v_{\text{lbl}},\;v_{{\text{syn}}_{1}},\;\ldots ,\;v_{{\text{syn}}_{k}}\}.$$

Each vector is stored in a Qdrant cluster [35] as:

{ ‘‘vector’’: [⋯ ],

 ‘‘payload’’: {‘‘text’’: ‘‘⋯’’,

 ‘‘url’’: ‘‘MONDO_ID or HP_ID’’ } }

preserving the ontology identifier as metadata for traceability.

For each outcome record in the dataset, the record text is embedded with the same model to produce a query vector q. The number of nearest neighbors to retrieve, k, is a user-defined hyperparameter. The top-k nearest neighbors are retrieved by cosine similarity:

$${\mathrm{sim}}_{\cos}(q,v)=\frac{q\cdot v}{\left||q||\;||v|\right|}.$$

For each query vector q, we form k candidate pairs $\{(q,\;v_{i}){\}}_{i=1}^{k}$, where $v_{i}$ denotes the i-th retrieved neighbor, each of which is subsequently evaluated by the LLM in the next step.

#### 2.4.2 SNOMED-based candidate generation

Healthcare organizations often use some form of internal classification or adopt ontology-based annotations. Hence, the input dataset may already be (partially) annotated, i.e., include helpful information besides natural language to relate the input entry to the target vocabulary. We explore a use case with patient EHRs classified according to the ICD-10 system (global classification of diseases).

The direct mapping of ICD-10 to MONDO/HPO is not available, with the exception of a small fraction of manually curated references from the MONDO ontology to ICD-10 codes. However, both systems can be linked via SNOMED CT which specifies hierarchical relationships connecting standardized codes for diseases with symptoms and procedures in EHR-based datasets.

A data pipeline to map ICD-10 codes to MONDO and HPO terms using SNOMED CT as an intermediary pivot is shown in Figure 2c. The technical implementation consists of three primary stages:

1. *ICD-10 to SNOMED CT Extraction*: The process begins by parsing the official SNOMED CT to ICD-10 extended map. It extracts the mapping between SNOMED CT identifiers and their corresponding ICD-10 targets. During extraction, ICD-10 codes are normalized by removing decimal points and auxiliary characters to ensure a uniform format for cross-system comparison.
2. *Ontology Cross-Reference Harvesting*: The pipeline processes the JSON-serialized graphs of the Mondo and HPO ontologies to harvest existing cross-references. It scans the metadata of each ontology node to identify cross-references tagged with SNOMED CT prefixes (specifically SCTID for Mondo and SNOMEDCT_US for HPO). These references are compiled into a lookup table where SNOMED identifiers are linked to the corresponding ontology URIs and clinical labels.
3. *Relational Join and Mapping Generation*: In the final stage, a relational join between the extracted ICD-10-to-SNOMED associations and the harvested SNOMED-to-Mondo/HPO cross-references is performed, linking ICD-10 codes to Mondo and/or HPO codes.

We form matching pairs as tuples $\left\{l_{i},\;m_{j}\right\}$ or $\left\{l_{i},\;h_{k}\right\}$ consisting of ICD-10 labels $l_{i}$ and MONDO labels $m_{i}$ or HPO labels $h_{k}$.

#### 2.4.3 LLM-based acceptance

The LLM (ChatGPT-4o) evaluates each tuple of pre-matched labels using the following acceptance prompt:

Given two short descriptions, decide whether they refer to the same disease or medical condition. If the second description is more narrow or specific, choose “No” as an answer. If the second description is broader or more generic, choose “Yes” as an answer. Start your answer from “Y” for “yes” or “N” for “no” and provide a concise justification, no more than 30 words, why you came to this conclusion.

## 3 Results

### 3.1 Unannotated dataset

The first dataset, provided by Kids First DRC [36], included 512 clinical records with pregnancy characteristic/risk factors, drug or chemical exposures, and outcome descriptions—without ontological annotations. We set k=3, yielding up to 3 candidate MONDO/HPO terms per input record, and producing in total 1,401 matching pairs.

**Figure 3:**
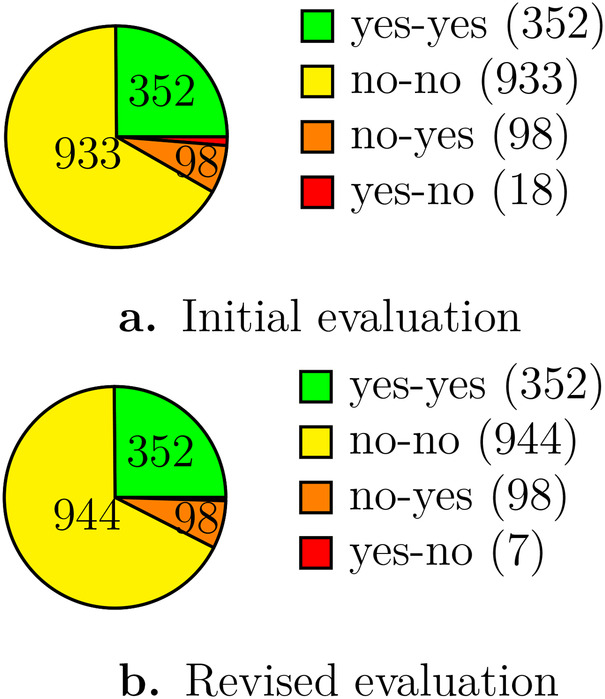
Number of mappings approved or rejected by Human vs. LLM. **(a)** Initial evaluation, **(b)** Revised evaluation.

To evaluate precision, a human expert (MD) independently assessed whether each matching pair referred to equivalent conditions as shown in Figure 3a. The LLM and expert agreed in 1,285 of 1,401 cases (92%). In 18 cases the expert accepted pairs the LLM rejected; in 98 cases the expert rejected pairs the LLM approved—of these, 57 involved a valid but more restrictive target description, and only 27 referred to genuinely different diseases.

The human expert was asked to review the decisions for pairs in which his assessment disagreed with the decision by the LLM. We clarified the requirements for the assessment of related outcomes similarly to the LLM prompt, asking to accept the mapping only if the target mapping is the same or more generic. The human expert retracted 11 out of 18 initially accepted mappings that he considered acceptable for the study context but that formally did not match the aforementioned relation. The revised results are shown in Figure 3b.

Tables 2, 3 give examples of EHR observations and suggested MONDO/HPO labels misjudged by the human expert and the LLM, respectively.

**Table 2:** Examples of mismatched conditions by LLM.

Patient record	Suggested mapping	Explanation
Trimester MOUD initiated—1st	Late first trimester onset	The first description mentions MOUD (Medications for Opioid Use Disorder), the second is about an unspecified condition observed toward the end of the first trimester.
Intraventricular hemorrhage > grade 2	Grade II preterm intraventricular haemorrhage	The first description refers to the grade above 2, the second to the grade 2.
Congenital laryngomalacia	Acquired laryngomalacia	Congenital condition is present at birth, acquired develops after birth.
APGAR score minute 1	10 min APGAR score of 1	The first description refers to the test performed at 1st minute after birth, the second at 10th minute.
Continued vomiting hours 1–4	Frequent vomiting	Vomiting persists over time without significant breaks as opposed to vomiting that happens many times.
Neonatal death	Neonatal lethal	The first description refers to the observed (confirmed) outcome, the second—to a severe condition likely causing death.
Respiratory	Respiratory infections	Respiratory problems are not necessarily caused by infections, the second description is more restrictive.
Loose stools	Frequent stools	The first description refers to consistency, the second to frequency (it may be normal in consistency but happens more often than normal)
Pharmacologic treatment of Nas	Nasal congestion	NAS (Neonatal Abstinence Syndrome) refers to the use of medications to manage withdrawal symptoms in newborns who have been exposed to opioids or other substances in utero.

**Table 3:** Examples of mismatched conditions by the human expert (revised after criteria clarification).

Patient record	Suggested mapping	Explanation
Severe intraventricular haemorrhage	Grade IV preterm intraventricular haemorrhage	Although the second description refers to a more specific condition, Grade IV implies that it is severe, hence the MD accepted this mapping.
Mechanical ventilation	Respiratory failure requiring assisted ventilation	The second description refers specifically to respiratory failure, whereas mechanical ventilation is a method of treatment. Since it is mentioned in the EHR, it implies the patient’s respiratory failure, hence the MD accepted this mapping.
Adverse events (AEs)	Adverse drug reaction (ADR)	AEs are any undesirable medical occurrences, not necessarily caused by the drug. Since our dataset describes outcomes of drug use in pregnancy, the MD assumed the AE is an ADR.
5 min Apgar <7	5 min APGAR score of 0	The first description is more generic.

### 3.2 Annotated dataset

The second dataset contained similar information but with outcomes pre-annotated using ICD-10 codes. The alignment task was therefore to translate 1,162 unique ICD-10 codes to corresponding MONDO and/or HPO terms. Direct identifier-based cross-references between these ontologies are sparse: only 1,840 MONDO and 39 HPO terms carry ICD-10 links (while HPO currently contains over 13,000 terms and over 156,000 annotations to hereditary diseases, MONDO defines approximately 25,600 disease terms, and the ICD-10 classification allows for more than 14,000 different codes). Hence, we used two complementary candidate-generation methods:

- **RAG-based:** vector search retrieving the 3 best-matching terms per ICD-10 code, producing 3,129 candidate pairs.
- **SNOMED-based:** connecting ICD-10 codes to MONDO/HPO via SNOMED CT cross-references, producing 7,787 candidate pairs. Because a single SNOMED concept may map to multiple ICD-10 codes [37], and appropriate codes can depend on patient-specific factors [38], LLM validation remains necessary.

**Figure 4:**
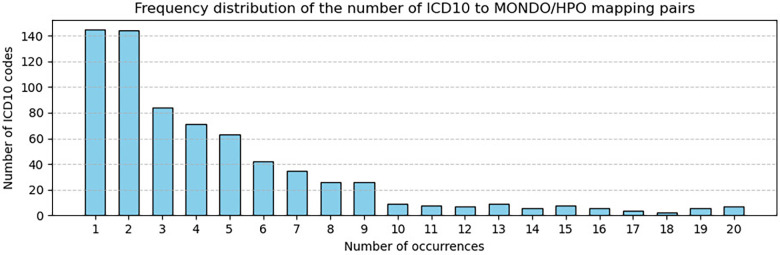
ICD-10-to-MONDO/HPO conversion via SNOMED, number of inputs vs target mappings.

The mapping method outlined in Figure 2c produced 7,787 matching pairs involving 800 original ICD-10 codes. For 362 ICD-10 codes no results were retrieved, either because (i) ICD-10 was not mentioned in SNOMED CT (192 cases) or (ii) SNOMED CT code was not mentioned in MONDO and HPO references (170 cases). Figure 4 shows part of the distribution of the number of relevant matches per input code retrieved via the SNOMED CT database. This distribution is extremely right-skewed; the image omits the entries that map into 20 or more codes. Although most of the ICD-10 codes (98%) map to 10 MONDO and HPO terms or less, 2% of ICD-10 conditions translate into 10 or more relevant MONDO and HPO terms, with extreme cases reaching over hundred of relevant mappings (see Table 4).

**Table 4:** Examples of ICD-10 codes with the large number of relevant MONDO and HPO terms.

ICD-10	ICD-10 label	*n*
Q878	Other specified congenital malformation syndromes, not elsewhere classified	329
Q870	Congenital malformation syndromes predominantly affecting facial appearance	287
Q828	Other specified congenital malformations of skin	195
Q788	Other specified osteochondrodysplasias	143
Q872	Congenital malformation syndromes predominantly involving limbs	136

**Figure 5:**
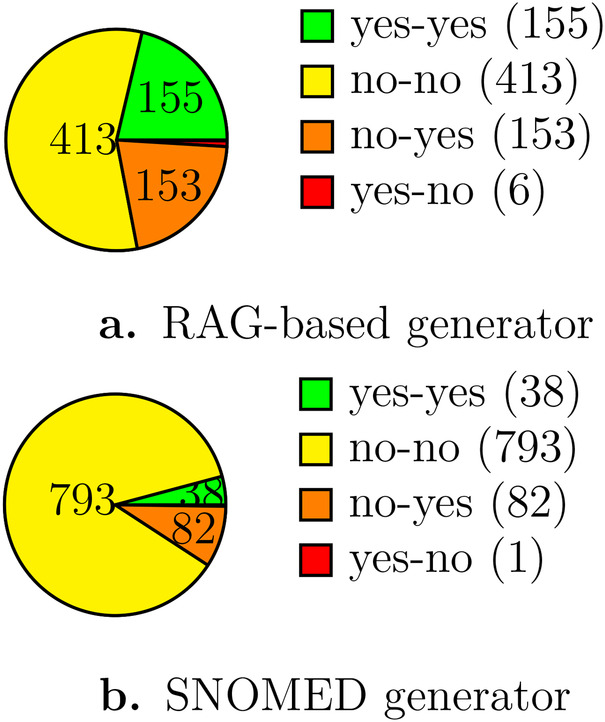
Number of mappings approved or rejected by Human vs. LLM. **(a)** RAG-based generator, **(b)** SNOMED generator.

In both cases, LLM was making the final decision whether to accept or reject the mappings, accepting pairs with an equivalent or more generic output. In the case of embedding-based matching, 42.3% of matching pairs were accepted. In the case of the SNOMED-based conversion, 14.7% of the matching pairs were accepted. Figure 5 shows the precision assessment of human expert vs. LLM for subsets (728 and 915 random records, respectively) of the records in both versions of ICD-10 to MONDO and HPO matching sets. In the RAG-generated pipeline, MD and LLM agree on 78% of decisions. With SNOMED-based matching pair generation, MD and LLM agree on 91% of the entries. The evaluation datasets and scripts to implement the presented mapping pipeline are available in [39].

For more relaxed acceptance criteria, i.e., whether both descriptions refer to the same disease or to the related but more general or more specific condition, the acceptance ratios were 71% and 80%, respectively.

## 4 Discussion

### 4.1 Performance of the two-step pipeline

The results demonstrate that embedding-based similarity alone is insufficient for reliable ontology mapping in clinical settings. Records in our datasets were often very short, contained abbreviations, or used ambiguous syntax, making vector-space retrieval an unreliable final arbiter. As Table 2 illustrates, superficially similar strings can refer to fundamentally different clinical concepts (e.g., *APGAR score minute 1* vs. *10 min APGAR score of 1*), errors that a nearest-neighbour search has no principled mechanism to catch.

The LLM validation step addresses this limitation directly. Expert—LLM agreement reached 92% on the unannotated dataset and 78%–91% on the annotated dataset depending on the candidate-generation method—accuracy comparable to the human expert while requiring no specialist time. Critically, the LLM acts as a semantic adjudicator: it reasons about ontological relations (equivalence, subsumption) rather than surface form, and rejects candidates whose embedding proximity is coincidental rather than conceptual. This is the core value of the two-step design: candidate generation maximizes recall cheaply, while LLM validation restores precision that embedding similarity alone cannot guarantee.

The LLM did show consistent difficulty with pairs that are semantically related but not equivalent, particularly cases involving specificity mismatches (e.g., severity gradations, congenital vs. acquired distinctions). This underscores the importance of explicitly encoding acceptance criteria in the prompt—specifically, whether the target term must be equivalent, more generic, or merely related—before deploying the pipeline in a new clinical context.

The two candidate-generation methods are complementary rather than interchangeable: only 475 of 1,162 ICD-10 codes were matched by both approaches, and neither alone achieved complete coverage. The SNOMED-based method failed to produce suggestions for 31% of codes, while the RAG-based pipeline’s recall was constrained by retrieving only three candidate terms (expanding to 10 would cover 98% of entries but substantially increases validation workload). Combining candidates from both generators and relying on the LLM to filter unsuitable mappings is therefore the most practical strategy for maximizing recall without sacrificing precision.

Taken together, these properties yield concrete advantages over manual and single-step automated approaches. Traditional expert-led harmonization requires a multidisciplinary team (clinician, ontology expert, data analyst) and produces non-reusable, project-specific artifacts. The two-step pipeline reduces this to a configurable, reusable recipe: overall mapping precision ranged from 78% to 92% across our experiments, with minimal human involvement. A library of such ready-to-use alignment functions between commonly used biomedical ontologies would be a durable resource for FL projects in healthcare.

### 4.2 Related work

Data harmonization in FL is complex due to varying formats, terminologies, and standards across decentralized data sources [40, 41]. In sensitive domains like pediatric care, privacy and consent requirements add further constraints [8, 42]. Stonebraker and Ilyas [43] highlight the limitations of traditional ETL pipelines and call for AI-assisted, declarative solutions to semantic heterogeneity—a gap our pipeline addresses directly.

Several recent works have explored LLMs for healthcare data interoperability. Li et al. [44] and Sett et al. [45] demonstrate that LLMs can map clinical text to FHIR standards with accuracy approaching human annotation. Santos et al. [46] and Matos et al. [47] show LLM-assisted harmonization of EHR schemata and concept extraction, both relying on top-k candidate matching followed by model-based selection—the same two-step logic we apply here. Fernandez et al. [48] predicted that LLMs would disrupt entity resolution and schema matching by enabling semantic understanding beyond surface similarity; our results corroborate this for ontology mapping. Wang et al. [49] caution that LLMs remain unreliable for complex diagnostic tasks; our use of LLMs as a binary semantic filter rather than a generative agent is deliberately conservative and consistent with this advice.

Privacy is a first-class concern for LLM-assisted pipelines in healthcare. Best-performing models are typically accessed via external APIs, which conflicts with data-protection policies in many clinical institutions. This motivates the use of open-source or locally hosted models, and the coupling of RAG with differential privacy [50]. Data heterogeneity and resource disparities across FL clients complicate model aggregation, reinforcing the need for robust upstream harmonization [3, 5, 42].

In contrast to domain-specific harmonization workflows [12], our work proposes a generalizable two-step recipe embedded in the Brane/EPI configurable execution environment. By treating LLMs as semantic adjudicators over ontology-indexed candidates, the approach is decoupled from any particular ontology pair or clinical domain and can be reused across FL projects without expert reconfiguration.

Currently, the best-performing LLM models are commonly accessed via API requests. This practice raises concerns about data privacy, and organizations with strict data protection policies, such as healthcare centers, are hesitant to adopt LLM-based pipelines. This motivates the need for better open-source models which are competitive with closed-source models. Another solution is to use private LLMs instances and/or couple RAG and LLMs with differential privacy solutions [50].

While existing frameworks and studies have made significant progress in addressing specific challenges in data harmonization for FL—ranging from schema alignment and semantic interoperability to privacy-preserving infrastructure and the integration of LLMs—most have been tailored to particular domains, use cases, or workflows. In contrast, our work on the Brane/EPI frameworks proposes a more generalizable approach: a configurable programming environment designed to support a wide range of research workflows. By introducing a generic recipe for ontology-based data mapping and leveraging LLMs as semantic adjudicators, we aim to enable scalable, interpretable, and ontology-aligned federated research across heterogeneous datasets.

### 4.3 Limitations and future work

The main limitation of this study is the evaluation setup: agreement was assessed by a single human expert, which may introduce bias. Broader validation involving multiple experts and inter-annotator agreement is needed to strengthen reliability.

Another practical limitation is deployment: demonstrating the federated Brane/EPI setup across institutions and datasets remains technically demanding. Future work will focus on simplifying deployment through a low-code FL environment within Brane/EPI, integrating data-agnostic AI components and model aggregation techniques into scientific workflows. We also plan to evaluate additional open-source LLMs under privacy-preserving conditions, and develop LLM-based assistants to streamline workflow definition, policy specification, and data alignment.

## 5 Conclusion

We introduced a two-step pipeline for ontology-based EHR harmonization: candidate term pairs are generated via vector-space retrieval or cross-reference traversal via several related ontologies, and an LLM acts as a semantic validator, accepting only ontologically consistent mappings. Across two real-world datasets, the method achieved 78%–92% agreement with expert assessment, with complementary retrieval strategies improving recall while preserving precision.

The approach reduces data harmonization from a manual, multidisciplinary effort to a reusable and configurable workflow. Importantly, embedding similarity alone is insufficient for reliable clinical ontology mapping; precision gains arise from the LLM’s ability to reason over equivalence and subsumption. This separation of recall (retrieval) and precision (LLM validation) is generalizable and can be applied to other source-to-target vocabulary mappings, including automated labeling, classification and selection of relevant patient cohorts, such as for clinical trial recruitment.

## Data Availability

The datasets presented in this study can be found in online repositories. The names of the repository/repositories and accession number(s) can be found below: https://zenodo.org/records/15411810.
